# Bone mineral properties and 3D orientation of human lamellar bone around cement lines and the Haversian system

**DOI:** 10.1107/S2052252523000866

**Published:** 2023-02-15

**Authors:** Tilman A. Grünewald, Andreas Johannes, Nina K. Wittig, Jonas Palle, Alexander Rack, Manfred Burghammer, Henrik Birkedal

**Affiliations:** a The European Synchrotron, Avenue des Martyrs 71, Grenoble 38000, France; b Aix-Marseille Université, CNRS, Centrale Marseille, Institut Fresnel, Marseille 13013, France; c Department of Chemistry and iNANO, Aarhus University, Gustav Wieds vej 14, Aarhus 8000, Denmark; UCL, United Kingdom

**Keywords:** human lamellar bone, X-ray scattering tensor tomography, Haversian system, cement lines, SAXS, WAXS

## Abstract

Bone is a complex, biological tissue made up primarily of collagen fibrils and biomineral nanoparticles. The importance of hierarchical organization in bone was realized early on, but the actual interplay between structural features and the properties on the nanostructural and crystallographic level is still a matter of intense discussion. Bone is the only mineralized tissue that can be remodeled and, at the start of the formation of new bone during this process, a structure called a cement line is formed on which regular bone grows. Here, the orientational relationship of nanostructural and crystallographic constituents as well as the structural properties of both nanostructural and crystallographic constituents around cement lines and the Haversian system in human lamellar bone are investigated. A combination of small- and wide-angle X-ray scattering tensor tomography is employed together with diffraction tomography and synchrotron computed tomography to generate a multi-modal image of the sample. This work shows that the mineral properties vary as a function of the distance to the Haversian canal and, importantly, shows that the cement line has differing mineral properties from the surrounding lamellar bone, in particular with respect to crystallite size and degree of orientation. Cement lines make up a significant portion of the bone matrix despite their small size, hence the reported findings on an altered mineral structure, together with the spatial modulation around the Haversian canal, have implications for the formation and mechanics of bone.

## Abstract 2

The hierarchical organization of bone is a key feature of this important biomineralized tissue. A combination of small- and wide-angle X-ray scattering tensor tomography with diffraction tomography and synchrotron computed tomography demonstrates how mineral properties and the orientational relationship between mineral and nanostructure vary across the tissue.

## 1. Introduction

The structure of bone is characterized by its hierarchical organization from millimetres down to the Ångstrom scale. Although the importance of this organization for the structural properties of bone has been recognized as early as the 19th century by Wolff, the actual make-up of bone, especially on the sub-micrometre length scale, is still under debate.

Bone is made from mineralized collagen fibrils, which are collagen fibrils mineralized with intra- and extrafibrillar hy­droxy­lapatite (HAP) mineral. The mineralized collagen fibrils are organized into various patterns of which lamellar organization is among the most important for mammals. In some species, including humans, the lamellar sheets are further organized into osteons which are characterized by a central blood vessel and a concentric succession of lamellar layers with varying fibrillar orientation. Individual osteons are delineated by a structure called a cement line, sometimes also referred to as a cement sheath (Raguin *et al.*, 2021 ▸). Although known since the late 19th century (von Ebner, 1875 ▸), little is known about its composition or function. In recent years, various attempts to characterize its mineral density or elemental composition led to reports of over (Langer *et al.*, 2012 ▸; Wittig *et al.*, 2019 ▸), under (Burr *et al.*, 1988 ▸) or normal mineral density (Raguin *et al.*, 2021 ▸) compared with surrounding bone tissue. Furthermore, it differs in its elemental composition (Pemmer *et al.*, 2013 ▸). An analysis of existing data indicated that cement lines have higher mineral density than surrounding bone (Wittig *et al.*, 2019 ▸).

Historically, the lack of knowledge stems mostly from the difficulty to characterize the bone structure at the sub-micrometre scale. Technical advancement like 3D FIB-SEM (Reznikov *et al.*, 2014 ▸; Raguin *et al.*, 2021 ▸) or high-resolution TEM tomography (McNally *et al.*, 2013 ▸; Reznikov *et al.*, 2018 ▸) have helped in our understanding of its organization on the crystallographic and nanostructural level. However, the downside of these techniques is a rather limited field of view when attaining high spatial resolution and invasive sample preparation (demineralization, staining), as well as sample modification by the focused ion beam. An alternative approach would be to employ X-ray diffraction, where the crystallographic texture can be determined very accurately in 2D (Grünewald *et al.*, 2016 ▸; Wagermaier *et al.*, 2007 ▸; Zhang *et al.*, 2020 ▸).

Tomography-based diffraction techniques have recently advanced from 2D to 3D studies (Wittig & Birkedal, 2022 ▸; Birkbak *et al.*, 2015 ▸). Here, the spatial resolution is limited by the X-ray beam size, typically in the range ∼1–10 µm, but in some instances spatial resolutions down to 120 nm have been reported (Palle *et al.*, 2020 ▸). The important point is that these techniques have excellent sensitivity to nanostructural and crystallographic properties from the diffraction signal (Zanghellini *et al.*, 2019 ▸; Wittig *et al.*, 2019 ▸; Fratzl-Zelman *et al.*, 2015 ▸) and, owing to the averaging of properties in the tomogram, they provide excellent sampling statistics. Recently, X-ray tensor tomography has enabled us to study the orientation of bone nanostructure (Liebi *et al.*, 2015 ▸; Schaff *et al.*, 2015 ▸; Liebi *et al.*, 2021 ▸) as well as bone mineral and nanostructure at the same time (Grünewald *et al.*, 2020 ▸), unveiling an orientational difference between the nanostructural and mineral orientation in human lamellar bone.

Diffraction and fluorescence tomography have shown that the microstructural properties of the mineral fraction in osteonal bone depend critically on the time point of formation. For example, the Sr content varies significantly as a function of mineral formation time as do also the crystallographic parameters of the HAP mineral fraction (Wittig *et al.*, 2019 ▸).

These findings prompted questions such as (i) what are the interrelations of the different structural motifs? (ii) What are the impacts of osteonal blood vessels, cement lines and lacunae on the structural properties? (iii) What is the orientational relation of structural motifs? To address these questions, we carried out SAXS/WAXS tensor tomography with a 2 µm beam coupled with phase-enhanced absorption synchrotron tomography with sub-micrometre spatial resolution on a human osteon in 3D.

## 2. Materials and methods

### 2.1. Sample preparation

An ∼100 µm cubed bone sample was extracted from femoral bone near the mid-diaphysis from a young male donor (Wittig *et al.*, 2019 ▸). First, an approximately 100 µm-thick slice of bone was cut parallel to the bone long axis, *i.e.* with the osteons running in the plane, using a diamond-blade saw (Accutom-5, M1D15 wheel, Struers, Ballerup, DK). An osteon was selected and cut with a standard razor blade through its center identified as the osteon canal. An additional cut approximately 100 µm from the first and parallel thereto was made, and from the resulting rod the final cuboid was cut. The sample was mounted on a drawn glass capillary (tip diameter ∼5 µm) with a small quantity of ep­oxy glue (Araldite Standard, Huntsman Advanced Materials, Basel, Switzerland).

### 2.2. Small/wide-angle scattering tensor tomography

Combined small-/wide-angle tensor tomography (SAXS/WAXS-TT) experiments (Grünewald *et al.*, 2020 ▸) were carried out at the ID13 beamline at the European Synchrotron Radiation Facility (ESRF), Grenoble, France. A 13 keV X-ray beam was defined using a channel-cut Si(111) monochromator with a bandwidth of ∼10^−4^. The beam was focused to a beam size of 2 × 2 µm with a set of beryllium compound refractive lenses. This configuration provided a flux of 2 × 10^12^ photons s^−1^ at the sample position. The sample was mounted on a goniometer composed of two SmarAct rotation stages, which was further motorized by a three-axis linear scanning stage. A 65 mm-long helium flight tube was inserted between the sample and a lead beam stop (Ø = 0.20 mm) to reduce air scattering. The small size of the beam stop precluded the addition of a photodiode and thus a second experiment was carried out to determine the absorption of the sample.

**Figure 1:**
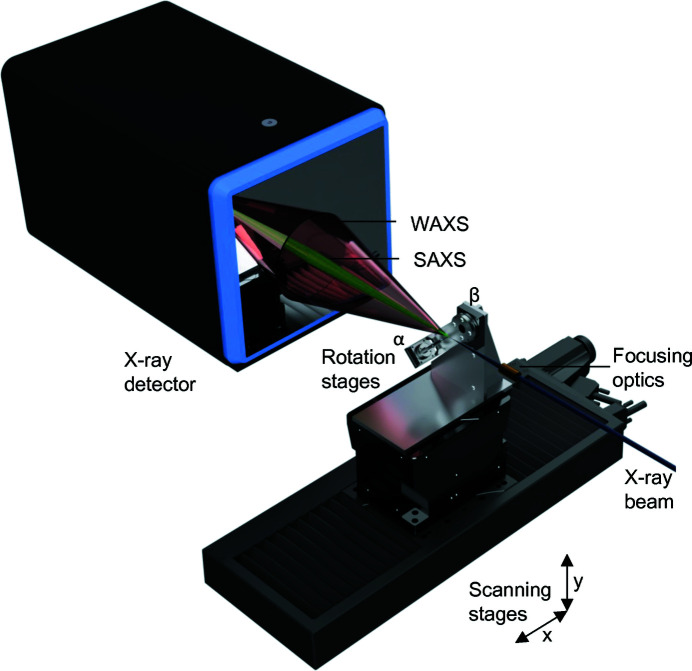
Experimental setup of SAXS/WAXS tensor tomography. An X-ray beam is focused on the sample by a set of compound refractive lenses. The sample is scanned through the beam by a set of *xy* scanning stages at various rotation and tilt angles around α and β. A full SAXS/WAXS pattern is collected at every point of the scan and used for the data reconstruction.

Scattering patterns were recorded using a Dectris Eiger 4M detector, placed 142 mm downstream of the sample. The setup gave access to scattering vectors (**q**) between 0.4 and 30 nm^−1^. A simplified schematic of the setup is given in Fig. 1 ▸.

A total of 288 projections were recorded at ten sample tilt angles (β) between 0 and 40° and rotation angles (α) between 0 and 180° (β = 0), and 0 and 360° (β ≠ 0). The rotational sampling was varied by a factor of cosβ to provide equal angular sampling in spherical 3D reciprocal space (Liebi *et al.*, 2018 ▸) and 70 projections were collected at β = 0.

The sample was pre-aligned at each angular position with an overview scan of 0.002 s exposure time, a step size of 20 µm and a field of view of 300 × 800 µm. The centroid of the total scattering signal was used to center the sample. Then, the sample was scanned with a step size of 2 µm and an exposure time of 0.01 s per point, with a field of view of 260 × 260 µm. All *x* *y* scans were carried out as fast scans (*i.e.* fly scans) with continuous movement of the fast (*x*) axis. An overview scan took 55 s, with 1.3 s of actual sample exposure time and the rest detector and movement overhead. The full scan took 302 s, with 171 s of actual sample exposure time. This gave a combined scan time of ∼27 h with an actual exposure time of 13 h. To put these numbers into perspective, the exposure time per voxel was 0.022 s. The X-ray dose (Howells *et al.*, 2009 ▸) *d* delivered to the sample was calculated as

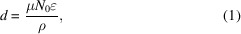

where μ is the linear absorption coefficient [54 × 10^2^ m^−1^ for Ca_5_(PO_4_)_3_OH at 13 keV (Hubbell & Seltzer, 1995 ▸)], *N*_0_ is the number of incident photons per unit area (1.4 × 10^20^ ph m^−2^), ɛ is the photon energy (2.08 × 10^−15^ J) and ρ is the mass density (2.5 g cm^−3^). Thus, the dose imparted on the sample for the full tensor tomogram was 6.48 × 10^9^ Gy. Under the assumption that each voxel absorbs the X-rays in a similar fashion, this equates to a dose of 2.95 × 10^3^ Gy per voxel.

### 2.3. Full-field synchrotron tomography

Full-field synchrotron microtomography data were collected on the ID19 beamline of the ESRF (Pacureanu *et al.*, 2012 ▸). The experiments were carried out using a pink beam, single-harmonic undulator source at 26.3 keV. A 1 mm diamond filter was added to the beam path. Projections were collected using a setup consisting of a 10 µm gadolinium–gallium garnet (GGG:Eu) scintillator and a high-resolution microscope (Optique Peter, France; Douissard *et al.*, 2012 ▸) with 10× lens and an sCMOS-based camera (pco.edge, PCO AG, Germany). This setup resulted in a voxel size of 650 nm. A total of 5000 projections were acquired with an exposure time of 0.065 s per projection. Data were reconstructed using the *pyHST* software package (Mirone *et al.*, 2014 ▸) with a filtered-back projection reconstruction algorithm.

### 2.4. Small-/wide-angle scattering tensor tomography reconstruction

The scattering data were reduced by integration in 16 azimuthal and 2000 radial bins. For the reconstruction of the SAXS signal, a *q*-range from 0.4 to 0.6 nm^−1^ was selected, and for the WAXS, the *q*-range corresponding to the HAP (002) reflection (*q* = 18.1–18.3 nm^−1^) was selected (Grünewald *et al.*, 2020 ▸). The data were normalized based on the air scattering surrounding the sample and the projections were aligned on the basis of their total SAXS scattering intensity signal. After a first alignment in the vertical direction based on the center of mass of each projection, the final alignment in both the vertical and the horizontal directions was carried out using the tomographic consistency approach (Guizar-Sicairos *et al.*, 2015 ▸).

The SAXS signal was reconstructed as an equatorial ring in the 3D reciprocal space representation, whereas the WAXS reconstruction was reconstructed as a signal on the poles of the 3D reciprocal space representation, owing to the orthogonality of SAXS and WAXS signals expected for the majority of bone (Grünewald *et al.*, 2020 ▸). Because of this alignment, the dot product of SAXS and WAXS orientation tensors is 1 for co-alignment. In order to account for X-ray absorption by the sample, the synchrotron CT was aligned to the SASTT scattering signal using the volume correlation function of the *Avizo* software package (version 8.1), and the absorption values obtained from absorption tomography were used for correction. The SASTT reconstruction can use the absorption in the forward direction for all azimuthal angles (Schroer *et al.*, 2006 ▸), whereas the WAXS signal needs to be corrected for absorption of the diffracted signal as a function of the azimuthal scattering angle. This was implemented by summing the absorption for each of the 16 angular segments for the *x*, *y*, β and α positions.

To this end, the linear absorption coefficient volume, determined by absorption tomography, was energy-corrected for the energy of the SAXS/WAXS-TT experiment. The scattered radiation passes through the sample at the diffraction angle 2θ defined as

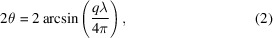

where *q* is the scattering vector and λ is the wavelength of the incident beam. It was assumed that the diffraction signal originates from the center of the sample thickness. Analogous to the tensor tomography reconstruction strategy, a projection operation can be employed to calculate the absorption (Liebi *et al.*, 2018 ▸, 2015 ▸) when 2θ and its trajectory towards the azimuthal detector segments are treated as an additional rotational offset around the tomographic axes α and β. With this approach, the transmission through the volume for each diffracted X-ray in each of the azimuthal detector segments can be easily calculated and the corresponding transmission can be used to correct the intensity of each detector segment prior to the WAXS tensor reconstruction.

A gradient-based optimization algorithm was used for the reconstruction, following the definition by Liebi *et al.* (2018 ▸). For the WAXS tensor reconstruction, the curvature of the Ewald sphere is taken into account as outlined by Grünewald *et al.* (2020 ▸).

The reconstructions were carried out with the code presented by Liebi *et al.* (2018 ▸), extended to account for the Ewald sphere curvature and WAXS absorption correction. The reconstructions were executed on a standard CPU server with 36 cores. The code took about 10 h to complete the full optimization. The extracted quantities of the degree of orientation (DOO) and orientation tensors follow previously published approaches (Liebi *et al.*, 2018 ▸).

### 2.5. Diffraction tomography data reconstruction and treatment

In order to obtain nanostructural and crystallographic information on the 3D volume, a filtered backprojection of the azimuthally averaged data at β = 0 was carried out, similar to that carried out by Schroer *et al.* (2006 ▸) for SAXS, and Stock *et al.* (2008 ▸) and Birkedal *et al.* (Leemreize *et al.*, 2013 ▸; Wittig *et al.*, 2019 ▸; Frølich *et al.*, 2016 ▸) for the diffraction signal. This corresponds to SAXS/WAXS-CT. Although it does not provide orientational information like SAXS/WAXSTT does, this approach yields a full 1D scattering curve for each voxel, which can be used to extract further structural information about the sample.

From the SAXS signal, assuming a two-phase system with a mineral fraction of 50% (Zizak *et al.*, 2003 ▸; Fratzl *et al.*, 1992 ▸) and predominately platelet-shaped particles, the mineral particle thickness (*T* parameter) was calculated using

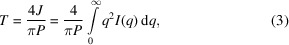

from the Porod constant *P* and the invariant *J*. The Porod constant was determined from the region *q* = 1 nm^−1^ to *q* = 3.5 nm^−1^, extracted from the data presented in a Porod plot, *Iq* ^4^ versus *q* ^4^. A linear fit was extrapolated to *q* = 0, which determines the Porod constant. The invariant *J* was determined from the Kratky plot, *Iq* ^2^ versus *q*, by integrating the scattering curve in the accessible *q* range and then extrapolating to *q* = 0 with *q* ^−4^ to *q* → ∞. This follows the approach laid out by Pabisch *et al.* (2013 ▸).

The crystalline lattice parameters can be extracted from the reconstructed WAXS signal. In bone, the mineral phase consists of hy­droxy­lapatite (HA)-like nanocrystals, which show a hexagonal structure, where the *c* axis and hence the scattering vector of the (002) reflection of the intrafibrillar mineral is considered to be oriented mostly in the long dimension of the mineral particles.

The width of the (002) reflection can be used to calculate the apparent mean length of the mineral crystals, whereas the position of the (002) reflection gives the lattice spacing in the direction of the *c* axis. The peak position and width were obtained by fitting a Gaussian function with a linear background to the reconstructed WAXS signal. The peak width was converted to an apparent crystallite size *L* using the Scherrer equation

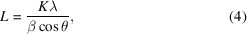

where *K* is the shape factor (0.94 in this case), λ is the X-ray wavelength, β is the peak width of the reflection after subtraction of the instrumental broadening [1.95 mrad for this setup (Zanghellini *et al.*, 2019 ▸)] and θ is the Bragg angle.

The absorption CT and SAXS/WAXS tensor tomography data were aligned volumetrically with the *Avizo* volume align functionality and transformed into a common coordinate system. From the absorption CT data, the osteocyte lacunae, the central blood vessel and the cement lines were segmented using a combination of Otsu thresholding and interactively defined grouping. Based on these segmentations, the parameters retrieved from SAXS/WAXS-CT were calculated as a function of the distance to the nearest structural feature, including the cement lines, blood vessel, lacunae and sample edges. The blue lines of Figs. 3 and 4 present the mean value of each distance group and the shaded areas delineate ±1 standard deviation.

In order to test for statistical significance of the difference between bone next to the various structural features and bone further away, the data presented in Figs. 3 and 4 were grouped into the first 10 µm and the last 10 µm and a one-way analysis of variance with a Bonferroni test was conducted [significance level = 0.05 (Liebi *et al.*, 2021 ▸)]. The two groups presented in Fig. 5 were similarly compared with a Bonferroni test at a significance level of 0.05.

## 3. Results

**Figure 2:**
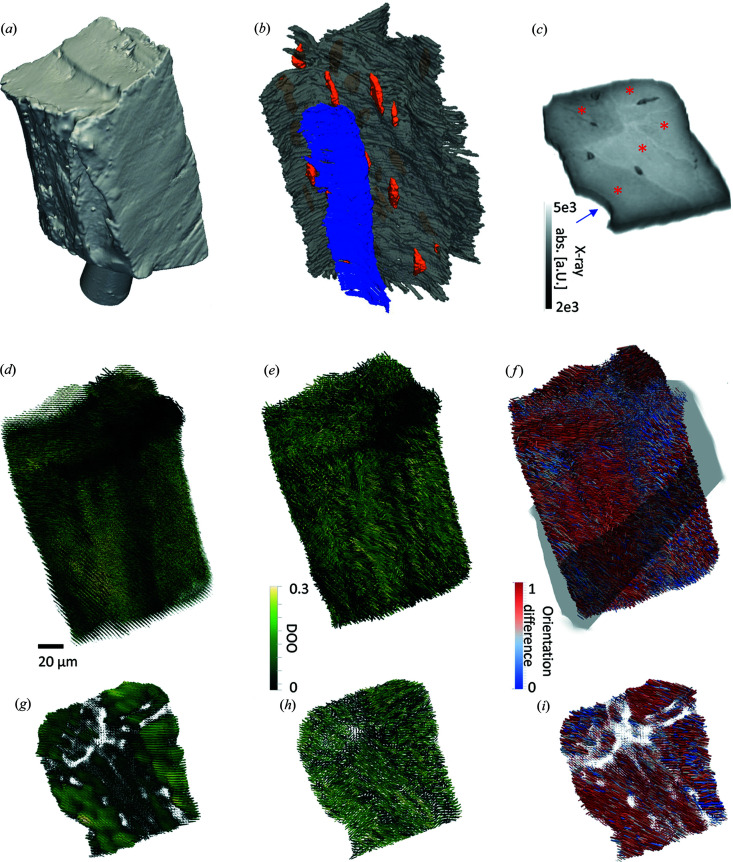
3D tomograms. (*a*) Surface rendering of the sample from synchrotron absorption tomography. (*b*) Segmentation of structural components based on the synchrotron absorption tomography data. Blue = blood vessel surface, orange = lacunae, gray = cement lines. (*c*) Slice through the absorption tomogram, showing the cement lines separating the different bone compartments (indicated by red asterisks) and the location of the central blood vessel (blue arrow). (*d*) SAXS tensor tomography. (*e*) WAXS tensor tomography. (*f*) Comparison of SAXS versus WAXS orientation, with the orientation difference calculated as the squared dot product color-coded with 1 (red) for co-orientation of mineral and nanostructure as in the classical models, and 0 (blue) for maximum orientation difference, gray outline indicates slice location for (*g*) and (*h*). (*g*) Slice through the SAXS orientation tomogram. (*h*) Slice through the WAXS orientation tomogram. (*i*) Slice through the SAXS versus WAXS orientation comparison tomogram.

We measured synchrotron CT [Figs. 2 ▸(*a*)–2 ▸(*c*)] and SAXS/WAXS-TT [Figs. 2 ▸(*d*) and 2 ▸(*e*)] in two separate experiments on the same specimen, covering a sample size of ∼100 × 100 × 200 µm. The sample consisted of a osteon with a portion of the central blood vessel visible and three separate bone compartments delineated by cement lines. The synchrotron CT is dominated by absorption effects and thus allows us to measure bone mineral density and identify the structural components, like the lacunar network (orange), blood vessel (blue) and cement lines (gray), rendered in Fig. 2 ▸(*b*) (see Methods[Sec sec2] for more details on the data treatment and segmentation). However, the absence of an absorption standard precludes further quantification of the bone mineral density. The synchrotron CT slice [Fig. 2 ▸(*c*)] shows the different anatomical features with the blood vessel in the lower left (blue arrow) and a strong contrast from the higher X-ray absorption of the cement lines delineating the different bone compartments (red asterisks). From tensor tomography, we obtain a diffraction curve for every voxel as well as an orientation tensor of the nanostructure from the SAXS signal [Fig. 2 ▸(*d*)] and the mineral particle from the HAP (002) WAXS signal [Fig. 2 ▸(*e*)], which is symbolized by a glyph pointing in the direction of the preferred orientation. The glyph length indicates the scattered symmetric intensity and the degree of orientation in every voxel is encoded in the color of the glyph in Figs. 2 ▸(*d*) and 2 ▸(*e*).

With direct access to the nanostructure [Fig. 2 ▸(*d*)] and crystallite [Fig. 2 ▸(*e*)] orientation tensors, it is possible to compare their orientational relationship by calculating the squared dot product of the two. In the classical models (Fratzl *et al.*, 2004 ▸; Landis, 1996 ▸), the mineral fraction and the collagen fibrils are strongly co-aligned. The HAP (002) reflection is aligned with the longest axis of the crystallites, whereas the SAXS signal stems from the thinnest dimensions of the mineral particles and to some extent also from the collagen fibrils. The orientations shown in Fig. 2 ▸ are the orientations of the scattering signal. The dot product of these two directions is thus 1 for a full co-alignment as predicted by the classical models, as indicated in red in Fig. 2 ▸(*f*) and the orientation difference is indicated in blue.

Figs. 2 ▸(*g*)–2 ▸(*i*) show two voxel-thick slices through the respective tomograms to further illustrate their orientations. The SAXS-TT slice shows that the different bone compartments exhibit varying degrees of orientation with the osteon near the blood vessel exhibiting the highest DOO. The shorter glyphs in the vicinity of the cement line indicate a weaker nanostructural scattering. Interestingly, this effect is less pronounced in the WAXS-TT dataset [Fig. 2 ▸(*h*)] while the same DOO trends are visible. The orientation difference between SAXS and WAXS is stronger around the cement lines and near the blood vessel [Fig. 2 ▸(*i*)].

**Figure 3:**
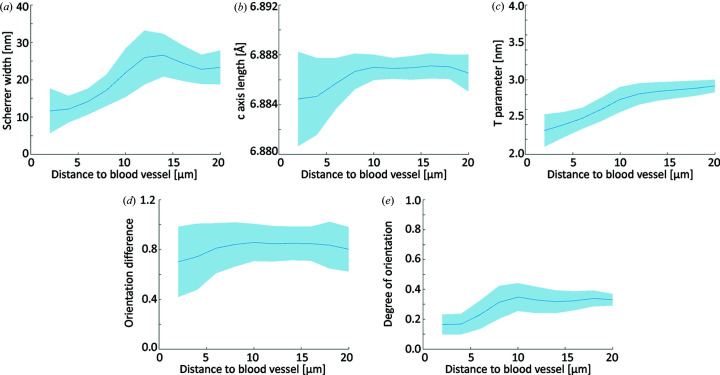
Nanostructural and crystallographic parameters plotted against the distance to the nearest blood vessel as determined by SAXS/WAXS-CT [panels (*a*)–(*c*)] and SAXS/WAXS-TT [panels (*d*) and (*e*)]. (*a*) Apparent crystallite size along the *c* axis (Scherrer width). (*b*) Length of the HAP *c* axis. (*c*) *T* parameter of the mineral particles. (*d*) Squared dot product of the SAXS and WAXS tensors. (*e*) Degree of orientation determined by SAXS-TT.

Fig. 3 ▸ shows the behavior of nanostructural and crystallographic properties as a function of the distance to the central blood vessel from the SAXS/WAXS-CT analyses. The mineral crystals display an increase in the (002) Scherrer width [Fig. 3 ▸(*a*)] as well as a slight lattice expansion [Fig. 3 ▸(*b*)] with increasing distance from the blood vessel. The mineral particle thickness likewise shows an increase with a larger distance to the blood vessel [Fig. 3 ▸(*c*)]. The size increase is greater within the first 10 µm and then levels off to a constant value. The dot product analysis of the SAXS/WAXS tensor orientation unveils a larger mismatch in orientation close to the blood vessel [Fig. 3 ▸(*d*)] and a generally lower degree of orientation is visible close the blood vessel [Fig. 3 ▸(*e*)]. All parameters showed significant differences when comparing the first and the last 10 µm from the blood vessel (*p* < 0.05). However, we would like to point out that the large number of voxels (observations) biases the *t*-statistics and renders them sensitive to differences, so care needs to be exercised in interpreting these results.

**Figure 4:**
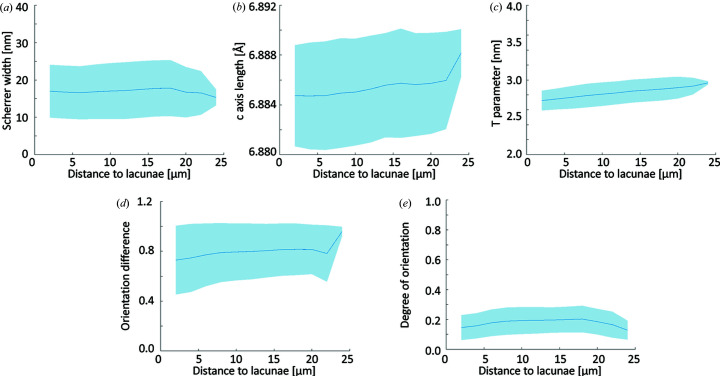
Nanostructural and crystallographic parameters plotted against the distance to the nearest lacunae as determined by SAXS/WAXS-CT [panels (*a*)–(*c*)] and SAXS/WAXS-TT [panels (*d*) and (*e*)]. (*a*) Apparent crystallite size (Scherrer width). (*b*) Length of the HAP *c* axis. (*c*) *T* parameter of the mineral particles. (*d*) Squared dot product of the SAXS and WAXS tensors. (*e*) Degree of orientation determined by SAXS-TT.

A similar distance analysis with respect to the osteocyte lacunae is presented in Fig. 4 ▸. Most mineral properties [Figs. 4 ▸(*a*) and 4 ▸(*b*)] show very little variation over the accessible distance. The mineral particle thickness [Fig. 4 ▸(*c*)] shows a slight and constant increase, comparable to the latter portion of Fig. 3 ▸(*c*). Both dot product [Fig. 4 ▸(*d*)] and degree of orientation [Fig. 4 ▸(*e*)] are slightly lowered in direct proximity to the lacunae. All parameters showed significant differences when comparing the first and the last 10 µm. We note that very few pixels contribute to the largest distances (24 µm). This renders them prone to noise and explains the differences observed at these large distances.

**Figure 5:**
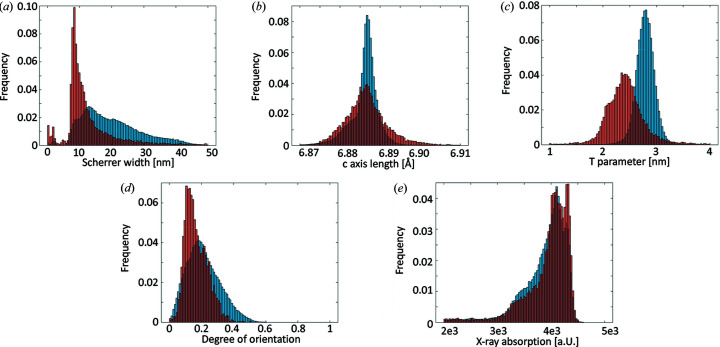
Histogram comparisons of cement line (red) and bone tissue (blue). (*a*) HAP (002) apparent crystallite size. (*b*) HAP *c* axis length. (*c*) *T* parameter. (*d*) Degree of orientation. (*e*) X-ray absorption.

Due to the low thickness of the cement lines and the associated uncertainty in their values owing to partial volume effects, we chose to isolate the cement line voxels (red) of the tomograms and compare them directly with the rest of the bone (blue) by means of a histogram analysis shown in Fig. 5 ▸. Note that the cement line voxels most likely also contain information from the surrounding bone due to the limited available resolution in the experiment. For the apparent crystal length, the two distributions are both asymmetric with tails towards larger lengths. Interestingly, they vary significantly in the apparent crystal length [Fig. 5 ▸(*a*)], with the cement line presenting a shorter apparent crystal length. The *c* axis length [Fig. 5 ▸(*b*)] differs slightly between the cement line and surrounding bone with the cement line voxels having a slightly contracted *c* axis. They also present a broadened distribution compared with the comparatively narrow histogram of the bone.

The *T* parameter determined from SAXS-CT [Fig. 5 ▸(*c*)] varies significantly between the two regions and the cement line presents markedly thinner mineral particles compared with the bone under the assumption that the *T* parameter can be interpreted as particle thickness. Note that the thickness interpretation requires a mineral volume fraction of 50% (Zizak *et al.*, 2003 ▸), a condition which is not necessarily met in the present case, leading to an overestimation of the mineral particle thickness for a mineralization below 50%. Though the trends shown in the *T* parameter are not altered by the mineral volume fraction, the actual values might differ. The degree of orientation [Fig. 5 ▸(*d*)] is slightly smaller in the cement line and, interestingly, the distribution is sharper than in the bone fraction. Finally, X-ray absorption is slightly higher and the distribution is slightly narrower for the cement line. Each parameter tested positive for statistically significant difference.

## 4. Discussion

### 4.1. Impact of microstructure on the mineral properties

The structural complexity of lamellar bone is underlined by the close proximity of different microstructural features like cement lines, blood vessels, canaliculi, osteons and bone compartments in the investigated bone volume as displayed in Fig. 2 ▸. It is of great importance to disentangle the influence of these structural features when it comes to the characterization of the bone mineral properties. It is a great strength of the employed methods SAXS/WAXS tensor tomography and absorption CT that they provide 3D volumetric data with good spatial resolution while providing sensitivity to the nanostructure and crystallographic structural properties with excellent sampling statistics.

The bone matrix close to the blood vessel is characterized by a different mineral structure with smaller crystallites, as indicated by the decrease in the Scherrer width [Fig. 3 ▸(*a*)] and a contracted *c* axis [Fig. 3 ▸(*b*)], potentially induced by lattice substitution or the signature of compressive strain. Recent research has shown that mineral properties are dependent on the time point of mineral formation (Wittig *et al.*, 2019 ▸), and in this paper, a similar behavior of the crystalline properties has been reported. In addition, we also report a smaller mineral particle size obtained from SAXS close to the blood vessel [Fig. 3 ▸(*c*)]. The values we find are consistent with those in the literature of 2.5 to 3.5 nm (Rinnerthaler *et al.*, 1999 ▸; Zanghellini *et al.*, 2019 ▸). The finding of small mineral particles during the early stages of growth, close to the blood vessel, supports the notion that varying the microenvironment does not only impact the crystal properties, but also the nanostructural size of the particles. We note that finding smaller crystallites as evaluated from the HAP (002) Scherrer width is consistent with smaller mineral particles.

In contrast to the changes visible in the vicinity of the blood vessel, we observe little change in the mineral structure properties in the vicinity of the osteocyte lacunae [Figs. 4 ▸(*a*) and 4 ▸(*b*)], except for a slight decrease in the mineral particle thickness close to the lacunae [Fig. 4 ▸(*c*)]. We explain this difference with the dominant role played by Haversian remodeling and the comparatively faster progression compared with the lacunar–canalicular bone remodeling. An additional factor to consider is that the structures under investigation are only two or three times larger than the spatial resolution of the experiment, introducing partial volume effects which additionally reduce the magnitude of any potential change.

### 4.2. Orientational relationship of the nanostructure and crystallographic structure

SAXS/WAXS tensor tomography is uniquely able to probe the 3D orientation of the nanostructure and mineral structure simultaneously and in a large sample volume. By analyzing the orientational relationship of the two, we can gain insights into the organization patterns in the bone.

The degree of orientation provides a straightforward way of evaluating anisotropy of the reconstructed scattering signal. In the present case, we analyze the SAXS degree of orientation. The degree of orientation is comparatively low at the blood vessel interface [Fig. 3 ▸(*e*)], indicating a rather disordered mineralized collagen fibril portion and is gaining in degree of orientation over the next ∼10 µm until it reaches a plateau. A qualitatively similar behavior can be observed around the lacunae [Fig. 4 ▸(*e*)], although we note that the variations are significantly less pronounced.

The dot product of the SAXS and WAXS orientation tensors compares the orientation of the SAXS nanostructure orientation with the HAP (002) orientation. A dot product of 1 indicates the co-alignment relation predicted by the classical bone models (Fratzl *et al.*, 2004 ▸; Landis, 1996 ▸), whereas a reduction indicates an alignment difference with respect to these models. Using this approach, we have recently shown (Grünewald *et al.*, 2020 ▸) localized orientation differences in human lamellar bone and interpreted them as a second, extrafibrillar mineral fraction. There is mounting evidence in the literature that a significant extrafibrillar mineral fraction exists (McNally *et al.*, 2013 ▸; Reznikov *et al.*, 2018 ▸; Bonar *et al.*, 1985 ▸), and may have important implications for the mechanical properties of bone (Hellmich & Ulm, 2002 ▸). An additional consideration here is the fact that the intrafibrillar channels in the collagen fibrils are tilted with respect to the fiber axis (Orgel *et al.*, 2006 ▸), and it was recently shown (Xu *et al.*, 2020 ▸) that these channels might direct the intrafibrillar mineral fraction under a tilt angle of about 5°, adding an additional contribution to the orientation difference.

In the present data, we observe a decreased dot product, *i.e.* less co-alignment, close to the central blood vessel [Fig. 3 ▸(*d*)] and, to a lesser extent, a reduction of the dot product close to the lacunar network [Fig. 4 ▸(*d*)]. At greater distances, the dot product reaches values of ∼0.85, in accordance with our previous experiments. In line with this, the reduction of co-alignment at the blood vessel interface can be interpreted as the more pronounced presence of an extrafibrillar mineral fraction, caused by the locally disordered collagen network which leaves more extrafibrillar space. This interpretation is supported by the findings of Raguin *et al.* (2021 ▸) who reported a thin disordered layer around osteocyte lacunae.

### 4.3. Structural characterization of the cement line

By extracting the voxels containing the cement line, we have a means of comparing the nanostructural and mineral structure therein with the surrounding bone matrix. Due to the strong heterogeneity of the surrounding bone matrix, we refrained from producing distance plots and chose to compare the cement line and the bone directly via histograms in order to judge the impact of the varying properties of bone. The histogram analysis presented in Fig. 5 ▸ shows some clear trends. On a crystallographic level, the cement line presents smaller crystallites as indicated by the Scherrer width [Fig. 5 ▸(*a*)], a slightly contracted *c* axis compared with the bone matrix [Fig. 5 ▸(*b*)], and presents significantly smaller mineral particles [Fig. 5 ▸(*c*)]. The nanostructural degree of orientation [Fig. 5 ▸(*d*)] is also lower and presents a narrower distribution compared with the bone matrix. This goes along with a higher mineral density of the cement line [Fig. 5 ▸(*e*)], as indicated by its higher X-ray absorption and is consistent with our previous observations in Wittig *et al.* (2019 ▸) and others (Langer *et al.*, 2012 ▸). In summary, it is evident that the crystalline material making up the cement line differs quite significantly from the surrounding bone matrix.

## 5. Conclusions

We used SAXS/WAXS tensor tomography and absorption tomography to characterize spatial variation of bone mineral properties and their orientational correlation to the bone nanostructure. We have shown that the mineral properties vary as a function of their anatomical location and those of the cement line differ significantly from those of surrounding lamellar bone with respect to their crystallite size and degree of orientation. These findings add a nanostructural and crystallographic dimension to the current discussion on the make-up of the cement line. We note that these surround all osteons in human bone and thus constitute a significant fraction of the bone even if each cement line is thin. Secondly, the thicker zone next to the blood vessel with altered orientational and crystallographic properties, as also reported by Wittig *et al.* (2019 ▸), indicates that the zone surrounding the osteon canal has a strongly altered biomineral organization, and a possibly differing fraction of extrafibrillar mineral and microstructure, which in turn has implications for bone mechanics and mineral homeostasis.
