# Bone mineral properties and 3D orientation of human lamellar bone around cement lines and the Haversian system. Corrigendum

**DOI:** 10.1107/S2052252523002695

**Published:** 2023-04-28

**Authors:** Tilman A. Grünewald, Andreas Johannes, Nina K. Wittig, Jonas Palle, Alexander Rack, Manfred Burghammer, Henrik Birkedal

**Affiliations:** a The European Synchrotron, Avenue des Martyrs 71, Grenoble 38000, France; b Aix-Marseille Université, CNRS, Centrale Marseille, Institut Fresnel, Marseille 13013, France; c Department of Chemistry and iNANO, Aarhus University, Gustav Wieds vej 14, Aarhus 8000, Denmark; UCL, United Kingdom

**Keywords:** human lamellar bone, X-ray scattering tensor tomography, Haversian system, cement lines, SAXS, WAXS

## Abstract

The article by Grünewald *et al.* [ *IUCrJ* (2023). **10**, 189–198] is corrected.

## Abstract 2

Corrigendum to the article by Grünewald *et al.* [ *IUCrJ* (2023). **10**, 189–198].

In the article by Grünewald *et al.* (2023 ▸), there was an error in the units in the calculation of the X-ray dose deposited. The units of *N*_0_ should read ph cm^−2^ and the reported value is indeed 1.4 × 10^20^ ph cm^−2^. The result of the dose calculation is not affected by this. The authors regret the confusion this may have caused.
